# Challenges and opportunities in patient-specific, motion-managed and PET/CT-guided radiation therapy of lung cancer: review and perspective

**DOI:** 10.1186/2001-1326-1-18

**Published:** 2012-08-31

**Authors:** Stephen R Bowen, Matthew J Nyflot, Michael Gensheimer, Kristi R G Hendrickson, Paul E Kinahan, George A Sandison, Shilpen A Patel

**Affiliations:** 1 University of Washington Medical Center, Department of Radiation Oncology, 1959 NE Pacific St, Box 356043, Seattle, WA 98195, USA; 2 University of Washington Medical Center, Department of Radiology, 1959 NE Pacific St, SS-202, Seattle, WA 98195, USA

**Keywords:** PET, FDG, Respiratory gating, Respiratory tracking, IMRT, IGRT, Adaptive radiotherapy

## Abstract

The increasing interest in combined positron emission tomography (PET) and computed tomography (CT) to guide lung cancer radiation therapy planning has been well documented. Motion management strategies during treatment simulation PET/CT imaging and treatment delivery have been proposed to improve the precision and accuracy of radiotherapy. In light of these research advances, why has translation of motion-managed PET/CT to clinical radiotherapy been slow and infrequent? Solutions to this problem are as complex as they are numerous, driven by large inter-patient variability in tumor motion trajectories across a highly heterogeneous population. Such variation dictates a comprehensive and patient-specific incorporation of motion management strategies into PET/CT-guided radiotherapy rather than a one-size-fits-all tactic. This review summarizes challenges and opportunities for clinical translation of advances in PET/CT-guided radiotherapy, as well as in respiratory motion-managed radiotherapy of lung cancer. These two concepts are then integrated into proposed patient-specific workflows that span classification schemes, PET/CT image formation, treatment planning, and adaptive image-guided radiotherapy delivery techniques.

## Review

### Introduction

Lung cancer is the leading cause of cancer mortality worldwide, resulting in 1.4 million deaths annually [1]. At the time of presentation, non-small cell lung cancer has often spread to multiple mediastinal lymph nodes and can no longer be successfully resected. Concurrent chemoradiation therapy is a mainstay of locally advanced lung cancer treatment, but standard-of-care regimens suffer from local failure rates as high as 85 percent for advanced stage non-small cell lung cancer patients [2]. Among other contributing factors, the potential efficacy of radiotherapy in these patients is compromised by uncertainty in lesion and normal tissue delineation due to respiratory-induced tumor motion, which has limited the precise planning and delivery of curative doses. Time-dependent computed tomography (CT) and positron emission tomography (PET) has the ability to resolve this motion and therefore define the extent of disease, both anatomically and functionally. In addition, the achievable therapeutic ratio of radiotherapy may be improved by image-guided dose intensification to PET-defined biological target volumes that are at highest risk of recurrence, and dose sparing of functional lung volumes that are at highest risk of complication. The application of motion-managed PET/CT to radiotherapy planning coupled with motion-managed and image-guided delivery will further individualize radiation oncology care of lung cancer patients.

**Figure 1:**
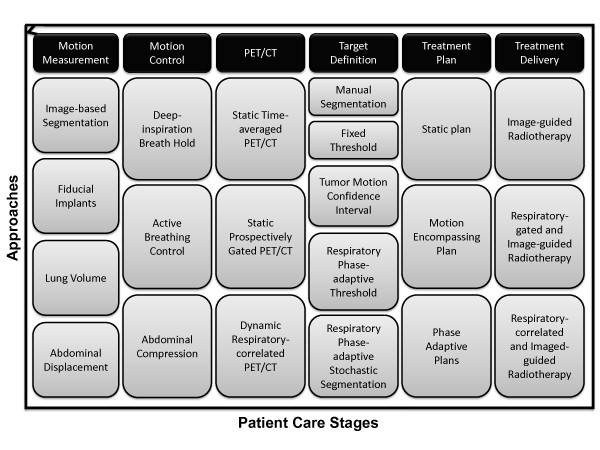
**Landscape of potential approaches to patient care in motion-managed and PET/CT-guided radiotherapy of lung cancer.** Across the stages of patient care, numerous approaches offer increasingly complex strategies. Details of each approach are given in Table 2.

Imaging with PET/CT is becoming a standard-of-care in the staging of lung cancers, but it is still underutilized in its direct integration to radiotherapy planning. Target volumes and uncertainty margins derived from PET imaging have been used without established consensus due to the complexity of the image formation process that yields quantitative radiotracer uptake information. Likewise, no consensus exists on optimal motion management techniques that can both reduce respiratory motion-induced image blurring and artifacts in PET/CT images of lung lesions as well as limit dosimetric errors during treatment planning and delivery. To address this multifaceted challenge, how does one best apply motion-managed PET/CT imaging to guide effective radiotherapy of lung cancer? Many clinical and technical tradeoffs must be accounted for between potential solutions, each of which may not benefit all individual patients within a typical heterogeneous treatment population. Table 1 summarizes the resulting gap between current clinical practice and potential application of research concepts, while Figure 1 illustrates the landscape of proposed approaches to various stages of lung cancer patient care in motion-managed and PET/CT-guided radiotherapy.

**Table 1:** Comparison of current clinical practice and research advances in motion-managed and PET/CT-guided radiotherapy of lung cancer

***Process***	***Clinical practice***	***Research advance***	***Challenge***	***Opportunity***
**PET/CT imaging**	Static PET/CT for diagnosis and staging	Respiratory motion-tracked PET/CT for treatment planning simulation	Precision and accuracy in PET/CT quantification	Standardization of PET/CT protocols
**Treatment planning**	Single plan from manual tumor segmentation with motion uncertainty margin	Adaptive plan from multi-phase tumor segmentation and biological target definition to maximize therapeutic ratio	Fast and reliable target definition from motion-managed PET/CT	Evaluation of potential therapeutic gains
**Treatment delivery**	Image-guided radiotherapy	Image-guided and motion-tracked radiotherapy	Adaptive motion tracking algorithm	Real-time verification of dose under motion management

A review of challenges and needed advances in PET/CT guidance and motion management of lung cancer radiotherapy is presented, followed by a perspective on translational strategies to make both guidance and management highly congruent to the individual patient.

### PET/CT guidance in lung cancer radiotherapy

While the clinical use of PET/CT in lung cancer patients is compelling, its application to radiotherapy has lagged somewhat and tends to be limited to qualitative assessment of disease extent or semi-quantitative tumor volume delineation as part of the treatment planning process [3,4]. If PET/CT treatment simulation becomes standardized, clinical translation of research advances in biological target definition and imaging uncertainty mitigation will follow.

#### Clinical usage

The most prevalent application of PET/CT to lung cancer patient care is diagnosis and staging using metabolic imaging of the radiotracer 2-deoxy-2-^18^ F]fluoro-D-glucose (FDG). In particular, the high sensitivity and specificity of FDG PET for the detection of involved regional lymph nodes and distant metastases [5] has resulted in the alteration of disease stage in a high percentage of lung cancer patients [6,7]. Though a topic of debate, some have even suggested that PET/CT imaging may reduce and eventually eliminate the need for more invasive diagnostic procedures such as mediastinoscopy, long considered the gold standard exam for lung cancer staging [8].

In addition to disease staging, FDG PET/CT is utilized in radiation therapy planning for gross tumor volume (GTV) and clinical target volume (CTV) definition [9,10]. PET/CT volumes are most often delineated manually by radiation oncologists, but may also be defined with quantitative techniques that operate on the standardized uptake value (SUV) in each image volume element (voxel). GTV segmentation based on PET/CT greatly reduces inter-observer variation relative to segmentation on CT alone [11-13] by discriminating between atelectasis, necrosis, and viable tumor. Additionally, PET/CT-defined tumor volumes achieve higher conformity to surgically resected and histopathologically defined volumes compared to those based on CT alone [14]. CTV definition is routinely altered by volume reduction from PET-negative lymph nodes and volume expansion from PET-positive nodes [5,7]. Overall target definition has been changed in an estimated range of 30 to 60 percent of patients [15]. As a result, the Radiation Therapy in Oncology Group (RTOG) 0515 has recommended standardizing the CTV definition of lung cancers by encompassing only the GTV and PET-positive lymph nodes [9].

Besides FDG PET, other ^18^ F-labeled radiotracers with potentially higher specificity towards imaging of particular molecular pathways have undergone or are in the process of clinical translation. These include but are not limited to surrogates of hypoxia using nitroimidazole compounds (e.g. ^18^ F]fluoromisonidazole [16]), cellular proliferation using thymidine analogs (e.g. ^18^ F]fluorothymidine [17,18]), osteablastic and osteoclastic activity using ^18^ F]NaF [19-21], and amino acid metabolism using tyrosine analogs (e.g. ^18^ F]fluoroethyltyrosine [22]). Though their role in guiding lung cancer radiotherapy is not yet established, the panel of imaging biomarkers represents a distinct trend towards personalized molecular profiling of patient disease to refine both staging and target definition. For example, the prognostic value of tumor hypoxia as evaluated by PET/CT in predicting poor clinical outcome has great potential for eventual clinical translation.

#### Current challenges

Clinical application of FDG PET/CT in lung cancer radiotherapy planning has been deterred by uncertainties in detection and quantification, which have led to difficulties in staging mediastinal involvement, regional lymph nodes, and distant metastases in certain cases [23]. Furthermore, the greatest source of uncertainty stems from a lack of consensus on tumor segmentation methods for target volume definition [24,25], particularly when comparing manual and simple threshold techniques [26]. This leads to large volume differences and poor correlation between some PET and CT-based target definitions [27,28]. Specifically, lung lesion shape and motion trajectory can shrink target volumes defined by absolute PET standardized uptake value (SUV) thresholds or inversely expand volumes defined by relative PET SUV thresholds [29]. Ground truth comparisons between PET/CT target volumes and pathological specimens are difficult without precise registration of the immunohistochemical fluorescence or autoradiographical image of the surgically resected sample and the segmented PET/CT image.

In addition to uncertainties in target delineation, precise and accurate quantification of PET/CT presents a challenge to radiotherapy planning of lung lesions. Tumor motion causes blurring of CT-derived anatomical electron densities and PET-derived activity concentrations, which can yield significant errors in the calculation of absorbed radiation doses from the former and in quantification of PET uptake from the latter [30,31]. These errors are compounded when attempting to correct for annihilation photon attenuation in PET from a CT image whose phase-sorted bins across the respiratory cycle do not properly match, resulting in positional errors that can exceed 10 mm [27].

#### Recent advances

Uncertainties in PET/CT-guided radiotherapy of lung cancer need to be mitigated by the implementation of robust target definitions and treatment planning algorithms. Improvements in PET/CT quantification include partial volume corrections methods to overcome limitations in PET spatial resolution, most commonly through sharpening of small image features with a point spread or imaging system response function [32]. PET images that more accurately reflect the underlying radiotracer distribution allow for more reliable automatic or objective tumor segmentation. Tumor volume definition algorithms range from motion-encompassing maximum intensity projections (MIP) [33], to linear regression of deterministic thresholds [34] and stochastic estimation of multivariate textural features. Recently, Hatt and colleagues have devised a fuzzy locally adaptive Bayesian (FLAB) segmentation algorithm [35]. The FLAB method relies on a probabilistic classification scheme of the PET image intensity distribution with fuzzy transitions between classes of voxels rather than discrete boundaries, which was shown to provide more robust metrics than summary statistics from simple SUV thresholds [36]. The most recent implementation of the algorithm, 3-FLAB, fits the image voxel uptake distribution into three classes: background, tumor, and tumor subvolume [37].

Once the radiotherapy target has been defined, treatment planning algorithms have emerged that can account for many sources of systematic and random errors, including those arising from motion-derived uncertainties during imaging of lung lesions. A class of objective functions, such as those found in the robust optimization package developed by Bortfeld and Unkelbach, allows for inputs in the form of probability density functions [38]. These functions calculate the relative likelihood that a given variable will have a particular value, which then places constraints on the planned dose distributions to reduce the overall variance from this source of uncertainty. Other investigators have constructed target coverage probability distributions from these uncertainties to estimate and correct for the propagated error in the planned dose distributions [39].

Advances in PET/CT-guided radiotherapy may enable the precise definition of lung cancer target volumes and prescriptions for biologically conformal delivery. Clinical trials investigating toxicity limits of PET/CT-based dose escalation to tumor subvolumes [40] include an upcoming RTOG study utilizing both pre-treatment and mid-treatment PET/CT-defined target volumes (RTOG 1105). However, the requisite level of quantification at sufficiently high spatial resolution may not allow for significant dose escalation to lung lesions without the explicit management of inter-fraction and intra-fraction motion.

### Respiratory motion management in lung cancer radiotherapy

Respiratory motion can be managed in one of the following two ways: motion *suppression* for static simulation imaging and treatment delivery, or motion *compensation* for dynamic simulation imaging and treatment delivery. An overview of motion management strategies is given here; for further details, please refer to the American Association of Physicists in Medicine Task Group 76 report [41].

#### Motion suppression

Respiratory motion suppression during treatment simulation imaging and treatment delivery is achieved either through breath hold or forced shallow breathing. Deep inspiration breath holds (DIBH) are used most commonly due to their clinical feasibility. DIBH has been shown to significantly decrease the lung density within the treatment field, thereby allowing tumor dose escalation without increasing late tissue complication risks in the form of pneumonitis or pulmonary edema [42]. However, intra-patient variation in inspiratory amplitude restricts the reproducibility of this procedure [43,44], which can be mitigated to varying degrees with effective audio-visual coaching of breathing techniques [45]. Residual motion during the breath hold can be measured with the aid of a respiratory surrogate marker.

Active breathing control (ABC) systems, including the commercial Active Breathing Coordinator™ (Elekta, Norcross, GA), were developed to permit more reproducible breath holds. ABC consists of a spirometer-controlled valve that can be set by the patient to close at a predefined lung volume, typically chosen between 50 and 80 percent of the maximum [46]. The patient controls the duration of the breath hold, making the process flexible to inter-patient and intra-patient variations in breathing patterns. Changes in absolute lung volume are assumed to be the primary cause of respiratory-induced tumor motion, meaning that control over this parameter can effectively reduce uncertainty in time-dependent tumor position by “freezing” the breathing state. Several requirements must be met under ABC-managed PET/CT imaging and treatment delivery: continuity of respiratory trace, patient suitability to achieve reproducible breath hold, and sufficient temporal efficiency. Spirometric measurement of lung volume relies on continuous changes in airflow within an airtight breathing apparatus, which has been shown to be susceptible to signal drifts [47]. Under circumstances when the seal on the ABC breathing tube mouthpiece temporarily breaks, or when the spirometer propeller rapidly changes angular direction at end-of-exhale, the respiratory trace may exhibit discontinuities that reduce the accuracy of the lung volume calculation. These errors are compounded by the inability of certain patients to consistently reach lung volumes that allow sufficiently large breath holds, typically at least 15 seconds. Short and infrequent breath holds reduce the temporal efficiency of both PET/CT imaging and treatment delivery, leading to protracted procedures that may no longer be clinically viable. ABC-based motion suppression is therefore indicated in patients with lung function that permits reproducible inspiration and the ability to frequently engage in a forced breath-hold [48].

Abdominal compression limits the amplitude of diaphragmatic respiration and thereby induces shallow chest breathing. It is commonly implemented with a frame-mounted chest plate, where the level of compression is controlled either through the position of a screw or from pneumatic pressure. Abdominal compression may be suited for lower lung lobe lesions near the diaphragm, where displacement of the abdominal surface is a strong correlate to tumor trajectory, but it is limited in suppressing motion of upper lung lobe lesions that are influenced by chest breathing. In a study on abdominal compression in 10 lung cancer patients, the inspiration-expiration tumor motion envelope, as measured on respiratory motion-tracked CT, was reduced from a mean of 13.6 mm to 8.3 mm and 7.2 mm under average forces of 47.6 N and 90.7 N, respectively [49]. The strength of this investigation was in the appropriate selection of patients with lower lobe lung and liver lesions for abdominal compression.

#### Motion compensation

The primary methods of motion compensation during treatment simulation imaging and treatment delivery exist: *gating* during a defined window of the respiratory cycle or *tracking* motion through correlation to the entire respiratory cycle. Both strategies require direct measures of tumor motion or indirect measures of the respiratory motion as a surrogate for tumor motion. Direct measures of target motion include implantable fiducial markers that are contrast enhancing in x-ray fluoroscopy image acquisition [50], wireless electromagnetic transponders and positron emission tomography (Xu et al. 2006). Indirect measures include, but are not limited to, external reflective optical or infrared markers of abdominal displacement [51], lung volume spirometer [47], or image segmentation of the diaphragm on x-ray projections combined with a lung motion model prior to CT reconstruction [52].

Respiratory gating typically bins the imaging acquisition or treatment delivery according to the direct or indirect measures of target motion amplitude [53,54], or alternatively according to the phase of the their periodic cycle [55]. As an example, the Real-time Position Management SystemTM (RPM) (Varian Medical Systems Inc., Palo Alto, CA) uses the abdominal displacement of infrared reflective markers to respiratory-gate both CT and PET image acquisitions on General Electric scanners, as well as treatment delivery [56]. Despite measuring identical markers of the respiratory signal, RPM-gated CT is accomplished retrospectively on triggers at peak inspiration in the RPM trace following image acquisition [45], whereas RPM-gated PET is achieved prospectively on the same peak inspiration triggers to define the gating cycle during image acquisition [57,58]. CT projections and PET coincidence events within the cycle are then sorted into bins according to a fixed percentage of the estimated breathing phase, typically 10 percent phases for CT and 20 percent phases for PET. While in principle one can choose an arbitrary gate during the breathing cycle for simulation imaging and treatment delivery, two techniques are used most frequently: end-of-exhale gating and peak-inhale gating. The end-of-exhale gating window features a longer dwell time during each breathing period and minimal residual tumor motion relative to other phases, whereas the peak-inhale gating window maximizes the lung volume to potentially increase separation between the tumor and neighboring critical structures.

Respiratory motion tracking uses similar external or internal markers as respiratory gating, but instead of specifying a finite window for imaging and treatment delivery, the tumor position is imaged over all breathing phases [59] and tracked during delivery in near real-time [60]. Assuming reliable correlation between the internal tumor motion and surrogate markers, the imaged trajectory at treatment simulation can be adaptively matched to the predicted trajectory during treatment delivery. Keall and colleagues first proposed the superposition of the respiratory motion pattern onto the planned radiation fluence map to allow for motion-tracked and intensity-modulated radiotherapy delivery [61]. They later validated this concept with dynamic multileaf collimator tracking [60,62].

#### Current challenges

Motion suppression techniques are prone to residual motion during treatment from hysteresis breathing patterns in the lateral and anterior-posterior dimensions, as well as over the course of treatment from daily setup errors. Patient coaching and tolerance for the motion suppression procedure strongly influences the degree of mitigation for residual tumor motion, meaning that selection of suitable candidates is of utmost importance. Management of these residual errors requires measurement from surrogate markers or on-board image guidance. Large residual errors, even under motion suppression, require respiratory-gated image acquisition and radiotherapy delivery for certain patients.

Respiratory gating efficacy is challenged by a tradeoff between motion blurring, image noise and treatment efficiency. For example, decreasing the respiratory-gated bin size will improve the temporal resolution of PET images to resolve tumor motion but at the cost of fewer detected coincidence events per bin and noisier images [58]. Respiratory-gated CT image sets are not usually noise-limited but are susceptible to artifacts from incorrectly sorted images based on a periodic motion. Treatment delivery over smaller gating windows reduces the intra-gate residual motion uncertainty but increases total delivery time substantially [63]. In particular, random drifts in absolute amplitude negatively affect end-of-exhale gating during imaging and treatment sessions, as well as between sessions.

Respiratory tracking using surrogate markers requires high and reproducible correlation to the internal tumor motion, which is patient-specific, imaging-specific, and radiotherapy fraction-specific. Furthermore, respiratory motion-tracked treatment delivery hinges on the accuracy and precision of tumor motion prediction algorithms to reposition the treatment couch or radiation beam at a particular time corresponding to a phase of the breathing cycle. For example, an adaptive filter algorithm can predict the position of a moving tumor 200 milliseconds into the future to overcome the latency of MLC movement, but estimation of this position carried an average uncertainty of 20 percent of the motion amplitude [60].

#### Recent advances

**Figure 2:**
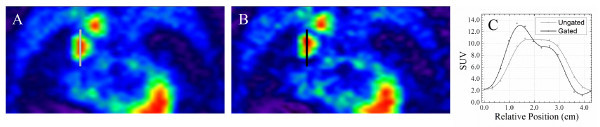
**Comparison of ungated (A) and quiescent period gated (B) [**^**18**^ **F]FDG PET image reconstructions.** The maximum standardized uptake value (SUV) is increased in the gated image of a detached lesion and the SUV profiles (**C**) show clear sharpening of the gated uptake spatial distribution (black line). This improvement in quantification would potentially alter the definition of biological targets for motion-compensated and PET/CT-guided radiotherapy.

Advances in respiratory gating include improved definitions for gating windows that allow for greater control over image noise/motion resolution tradeoffs, reproducible gated delivery and clinically efficient procedures. Liu and colleagues developed a quiescent period PET gating algorithm that retrospectively bins an RPM displacement histogram based on the percentage of total counts within the gating window centered on the mode of the distribution [64]. Alternatively, they proposed prospectively gating during a quiescent period based on a percentage of peak inspiration for each breathing cycle, which accounts for inter-cycle variations and minimizes drifts in the average position over time (see Figure 2). Furthermore, improved quantification of gated PET/CT from accurate CT-based attenuation correction is accomplished by correlating the internal trajectory of tumor centroids to the RPM block displacement [65]. Such regression analysis is critical to calculate the relationship between respiratory marker time-dependent positions as surrogates for tumor time-dependent positions in each individual patient. In general, correlation studies between measures of external respiratory motion surrogates and internal tumor motion have reported linear relationships in the superior-inferior dimension of motion but poorer agreement in patients with significant hysteresis or a lesion attached to another structure, since they may exhibit more chaotic trajectories [66].

Advances in respiratory tracking have increased near real-time tumor motion prediction [67]. Two classes of algorithms have emerged as leading candidates for clinical application: (periodic) auto-regressive moving average (PARMA) and support vector machine (SVM) learning, both of which adapt to drifts and random components superposed on the sinusoidal signal. The ARMA [68] and PARMA [69] methods realize up to 500-ms motion prediction at the 95 percent confidence interval, while SVM-based methods achieved 2-mm root-mean-square error at 1-s motion prediction [70]. Predicted tumor trajectories can then be programmed into robotic couches with fully three-dimensional translational and rotational degrees of freedom to ensure that the radiation beam continuously conforms to its intended target [71-73]. For example, the SynchronyTM respiratory tracking solution (Accuray, Sunnyvale, CA) adapts to changes in breathing patterns through repeat x-ray projections that update the correlative relationship between internal target motion and external marker motion [74]. Alternatively, the treatment couch can remain stationary and the predicted trajectories are then programmed into the multileaf collimator to actively reshape the radiation beam to conform to the moving target [75]. These two approaches to respiratory-tracked radiotherapy delivery are implemented from differing frames of reference: the couch in the patient’s coordinate system, or the collimator in the beam’s coordinate system. Couch and beam tracking may be eventually combined so as to mitigate errors in couch displacement, reported to range from 0.1-0.3 mm [72], and collimator leaf displacement limits.

### Personalized motion-managed and PET/CT-guided radiotherapy workflows

Given the high degree of variability between lung cancer patients, clearly some may be better suited for motion-suppressed radiotherapy while other may benefit from motion-compensated radiotherapy. The advantages and disadvantages of various motion-managed and PET/CT-guided radiotherapy approaches are listed in Table 2. The systematic integration of these approaches to form patient-specific clinical workflows has great potential to improve accuracy of treatment planning and delivery. The workflows are characterized by many distinct components: patient classification, PET/CT image acquisition and reconstruction, target and prescription definition, radiotherapy planning, treatment plan quality assurance, image-guided radiotherapy delivery, and adaptive procedures. Each workflow component section concludes with a translational question to the clinical and scientific communities for further investigation, whose answers will rely on strong interdisciplinary collaboration.

**Table 2:** Motion-managed and PET/CT-guided radiotherapy components

***Approaches***	***Advantages***	***Disadvantages***	***Comments***
Abdominal displacement markers	Clinical feasibility	Insensitive to small abdominal displacements	Indicated for most patients. Use patient-specific block position, camera aperture and brightness to maximize detectable abdominal displacement
Lung volume spirometer	Stronger correlation to internal target motion	Patient coaching complexity	Indicated in patients with small abdominal displacements
Fiducial implants	Direct image of internal target motion	Invasive procedure and subsequent migration	Indicated in patients with accessible lesions when other respiratory signal surrogates not indicated
Image segmentation of diaphragm ROI	Non-invasive measure of respiratory motion	Challenges associated with deformable registration across phases	Ensure phase-sorted images not undersampled through sufficient projections or reliable undersampled image reconstruction algorithms
Deep inspiration breath hold	Clinical feasibility	Lack of reproducibility and temporal inefficiency	Indicated in patients with sufficient lung function to allow for reliable breath hold under audiovisual coaching
Active Breathing Control	Reduction of motion envelope	Lung function requirement to permit forced breath hold	Determine patient-specific lung volume for breath hold (50–80% of max)
Abdominal compression	Reduction of abdominal displacement	Upper lobe lesions subject to motion in non-diaphragmatic breathers	Indicated in diaphragmatic breathers with additional measurement of residual motion when possible to enact tolerance criteria
Static PET/CT	Reproducibility	Motion-blurred image	Indicated for low amplitude motion lesions (e.g. upper lobe, chest wall attached)
Static prospectively gated PET/CT	Suppression of motion blurring without loss of SNR	Temporal inefficiency	Use in conjunction with ABC for patients with random breathing pattern that can achieve sufficient lung volume
Dynamic motion-tracked PET/CT	Better representation of target motion	Challenge to reproduce correlation at treatment	Use in conjuction with RF block, spirometer, fiducials, or image segmentation over all phases of breathing cycle for patients with periodic breathing
Phase-averaged PET/CT	Robust low noise image	Reduced contrast and quantitative accuracy without motion information	Evaluate helical CT to determine whether to use phase-averaged PET or motion-compensated PET/CT
Maximum Intensity Projection PET/CT	Represents high confidence interval of motion envelope	PET image SNR reduced to equivalent counts for single phase	Weight intensity projection distribution across respiratory phases to improve SNR while maintaining motion envelope confidence interval
Quiescent period gated PET/CT	Variance reduction from motion over reproducible phase bin	Image quality dependent on fractional counts within quiescent window	Patient-specific gating window based on either relative displacement amplitude or absolute phase
Multiphase PET/CT	Motion compensated images with little information loss	Requires sufficient correlation between respiratory signal and target motion	Optimize number of phases and phase bin sizes as function of lesion size, location, motion amplitude
Manual contour	Patient-specific target delineation	Inter-observer variability in target definition	Useful as higher order correction to target definition following automated techniques
Absolute/relative threshold	Clinical feasibility	Uncertainty in threshold due to noise or variation in backround uptake	Validate threshold-defined targets as prognostic factors of treatment outcome in abdominothoracic cancer patients
Confidence interval	Target motion margins weighted by spatiotemporal likelihood map	Limited to single target envelope by ignoring phase-specific information	Establish relevant confidence interval criteria based on MIP or motion-weighted intensity projection to build dose volume relationship for fixed normal tissue integral dose
Phase adaptive threshold	ROI specific to different phases of target motion	Complexity of threshold determination for all phases	Validate phase-adapted threshold-defined targets against known target parameters in motion phantoms
Phase adaptive stochastic segmentation	Robust to image noise and heterogeneities	Dependent on initialization conditions and susceptible to statistical variation	Validate in motion phantoms followed by comparison of prognostic value to phase-averaged targets
Single plan from ROI	Clinical feasibility	Single plan may require frequent adaptation during treatment course	Indicated in patients with fewer normal tissue tolerance constraints that allow for sufficient target dose
Single plan from optimal margin target definition	Single plan feasibility with motion-compensated target definition	Reduced delivery degrees of freedom compared to phase-adapted plan	Indicated in patients whose single plan normal tissue constraints do not allow for sufficient target dose
Phase-adapted plan	Physical/biological advantages to differential delivery across phases	No consensus on weighting scheme for phase fluence maps	Indicated in patients whose single motion-compensated plan normal tissue constraints do not allow for sufficient target dose
Single plan to static phantom	Clinical feasibility	Ignores impact of motion on clinical deliverability of treatment plan	Baseline measure of plan deliverability prior to motion uncertainties
Single plan to patient-specific motion phantom	Accounts for realistic motion trajectories	Plan deliverability limited by motion	Plans that fail QA due to motion should be replanned on individual phases
Phase-adapted plan to patient-specific motion phantom	Characterize deliverability of phase-correlated plan	Higher sensitivity to phantom setup and dosimeter measurement uncertainties	Ensure precise and accurate setup of phantom and sufficient spatiotemporal resolution of dosimeters
IGRT	Clinical feasibility	Reliant on motion control or static lesion to maximize delivery efficacy	Daily imaging to verify target motion envelope within PTV
Respiratory-gated IGRT	Compromise between delivery reproducibility and treatment efficacy	Temporal inefficiency	Ensure gating window provides sufficient target coverage to phase gate-matched PTV through daily imaging and respiratory signal measurement
Respiratory-tracked IGRT	Advanced delivery optimized to complete target motion trajectory	Requires accurate and precise motion prediction algorithm to account for delivery system latency	Ensure correlation between imaged target trajectory and planned phase-correlated target trajectory
Planned adaptive treatment	Adapt to morphological and biological changes during RT	Adapted plan does not account for changes in image signal due to motion	Establish criteria for adapting plan that include uncertainties in imaging signal change due to motion
Planned phase-adaptive treatment	Adapt to motion-compensated morphological and biological changes during RT	Challenge of re-planning from mid Tx motion-compensated PET/CT or from on-board imager alone	Determine disease and site-specific criteria for adapting plan based on PET/CT or on-board imager

#### Patient classification

**Figure 3:**
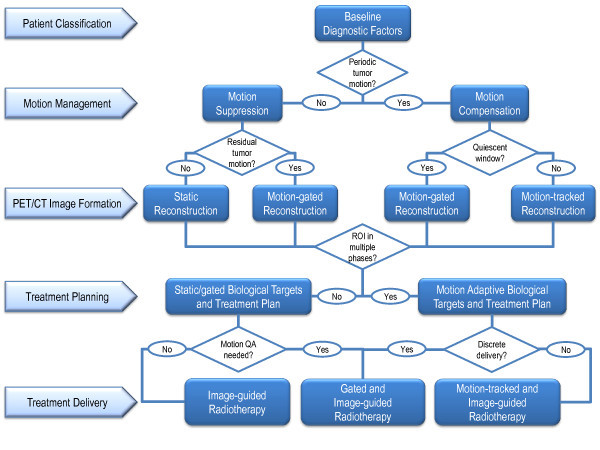
**Example workflow of patient-specific motion management and PET/CT guidance for lung cancer radiotherapy.** Beginning with patient classification based on diagnostic factors, motion is either suppressed or compensated for during the PET/CT acquisition. Static, respiratory-gated, or respiratory motion-tracked images are then used to define biological targets for treatment plans. Radiotherapy is delivered under image guidance when motion is suppressed, during a particular respiratory gate that is matched to the plan or throughout the respiratory cycle by predictively tracking the motion.

Due to the high degree of heterogeneity in the lung cancer patient population, including variations in respiratory-induced tumor motion, personalized motion management of PET/CT-guided radiotherapy begins with intelligent patient classification. Various forms of classification have arisen across several biomedical disciplines, most notably genomics, proteomics, and metabolomics, to account for cancer-specific variations within large bioinformatic datasets. A similar approach can be applied to develop hierarchical clustering models of lung cancer patient populations according to respiratory parameters and diagnostic imaging metrics. One example patient-specific workflow, shown schematically in Figure 3, uses several decision criteria to triage prospective patients into different imaging, treatment planning, and treatment delivery protocols. The objective would be to integrate patient-specific information in order to maximize the achievable quantitative accuracy and precision throughout a motion managed and PET/CT radiotherapy workflow.

Signal processing of respiratory traces has revealed that lung cancer patients can be grouped into three broad categories: 60 percent of patients can be classified as periodic breathers with reproducible end-expiration displacement, 20 percent as periodic breathers with normal distributions of end-expiration displacement, and 20 percent as chaotic breathers [29]. In addition to spectral analysis of respiratory patterns, baseline diagnostic imaging factors from CT (lesion size and location), as well as those from available PET studies (FDG avidity and uptake spatial heterogeneity), could refine the definition of these patient classes. The classes should account for a high percentage of the inter-patient variability that is likely to impact the motion management technique during PET/CT treatment simulation, radiotherapy planning and delivery.

Given this set of baseline parameters, patients could, for example, be stratified to several cohorts: (A) respiratory motion-gated PET/CT and radiotherapy, (B) respiratory motion-tracked PET/CT and radiotherapy, or (C) motion-suppressed PET/CT and radiotherapy. In general, the most complex treatment simulation and delivery should be reserved for patients with reproducible periodic tumor motion that can be compensated for with high precision and accuracy. Following this classification scheme, patients with long end-expiration breathing pattern could follow Cohort A with a quiescent period RPM-gated PET/CT imaging protocol with phase-matched 3D conformal or intensity-modulated radiotherapy delivery. Those with focal lesions, homogeneous FDG avidity, and periodic breathing patterns could follow Cohort B with a respiratory motion-tracked PET/CT and predictive phase-optimized volumetrically modulated arc therapy delivery. Finally, those with diffuse lesions, mediastinum or chest wall attachment, heterogeneous FDG avidity, and chaotic breathing patterns could follow Cohort C with active breathing control or abdominal compression during PET/CT-guided radiotherapy. This would result in three sets of patients who would undergo dramatically different motion-managed and PET/CT-guided radiotherapy regimens.

**Figure 4:**
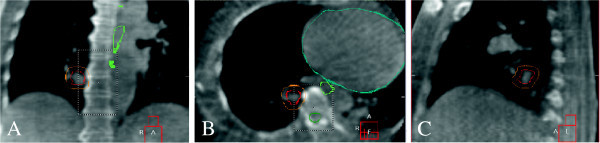
**Four-dimensional cone beam computed tomography for image-guided radiotherapy.** Images acquired at the time of treatment delivery are sorted into temporal phases according to the time-dependent diaphragm position. Coronal (**A**), transaxial (**B**) and sagittal (**C**) views at 30 percent phase show the gross tumor volume (red contour), planning target volume (orange contour) and esophagus (green contour). The PTV was defined on the maximum intensity projection of a respiratory-gated simulation CT. While the PTV encompasses the motion of the lesion prior to treatment delivery, its definition may be enhanced through individual phase adaption as part of a motion-managed and PET/CT-guided radiotherapy regimen.

Certain assumptions and caveats apply. For instance, contingencies must be in place in the event that a given external respiratory motion surrogate (i.e. RPM block, ABC spirometer, etc.) does not correlate with the internal tumor trajectory. In this case, respiratory gating or tracking may be better achieved with direct imaging of the internal motion, as measured by implanted fiducial markers or diaphragm image segmentation (see Figure 4). More broadly speaking, not all individual patients may fall within one of the classification categories due to the continuous distribution of the imaging and respiratory signal parameters across the patient population. It is important to quantify residual errors that ensue from each motion management strategy, which would then be propagated through the simulation imaging, treatment planning, and treatment delivery processes. In this manner, uncertainties in target volumes and quality assurance tolerances could be determined for individual patients and incorporated via spatial and dosimetric margins within the PET/CT-guided treatment plan.

How do we effectively translate the vast array of patient classifiers to a clinically meaningful and implementable selection algorithm?

#### PET/CT image acquisition and reconstruction

Three types of PET/CT image acquisition can be performed either under free-breathing or breath-hold conditions: static, gated, or correlated acquisitions. Static acquisitions utilize all image projections without any respiratory motion information from which to sort the data. Gated acquisitions set triggers to bin data within a particular window (e.g. peak inspiration or end-of-expiration) that defines a breathing state. Correlated acquisitions sort all image projections into differing respiratory states to image the complete time-dependent tumor position.

In cases where tumor motion carries an uncertainty that is significantly smaller than other sources of error in the image formation process, either with or without motion suppression, then simple static whole body PET/CT acquisitions are indicated. Clinical PET scanners have an average image spatial resolution of 5 mm, which implies that detectable changes in activity concentration distribution from the complete resolution of tumor motion alone would need to arise from amplitudes of at least 10 mm according to the Shannon-Nyquist sampling criterion. Patients with upper lobe lesions that are tethered to the mediastinum or outer chest wall are likely to have small motion envelopes characterized by a complex and highly deformable trajectory. Therefore, static PET/CT acquisitions under active breathing controlled breath holds or abdominal compression should in principle result in the least variability in quantification of image intensity values in these patients, but at the cost of more difficult clinical feasibility due to challenges in patient coaching and tolerability.

At the other end of the spectrum, cases with tumor motion-derived uncertainty exceeding 10 mm in sinusoidal amplitude call for respiratory motion-tracked PET/CT. CT acquisition in cine mode is followed by PET acquisition of the list mode coincidence events, which for example can be both RPM-sorted into matching phases of the breathing cycle. This requires correlation of the external RPM block position with the internal tumor position on a patient-specific basis for PET attenuation correction. Correlation of the external respiratory surrogate and internal tumor motion trajectories can be verified independently from diaphragm segmentation-sorted CT images.

**Figure 5:**
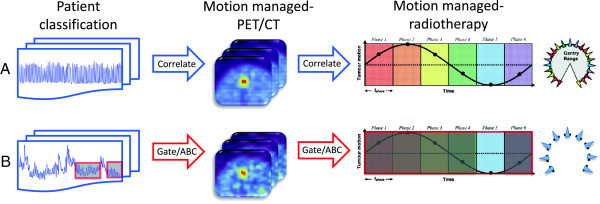
**Examples of motion management strategies for two patients receiving PET/CT-guided radiotherapy.** The top row de s a patient whose baseline diagnostic factors indicate a highly period tumor motion and respiratory pattern, suitable for respiratory motion-tracked PET/CT and motion-tracked radiotherapy. The bottom row illustrates a patient whose chaotic respiratory pattern makes them suitable for PET/CT and radiotherapy under active breathing control (**ABC**) and prospective respiratory gating during a finite time period. Figure adapted from Liu *et al.* and Chin *et al.*[29,65,76].

Respiratory gating can be accomplished either prospectively or retrospectively in patients that present with viable gating windows. Patients with pronounced quiescent peaks at end-expiration could undergo retrospectively gated PET/CT acquisitions that balance image noise and motion resolution tradeoffs, as shown in Figure 5A. On the other hand, patients with drifts in the quiescent period at end-expiration may benefit from prospectively gated PET/CT acquisitions that adapt to inter-cycle variations in motion amplitude and moving averages. Respiratory gating may not be suitable for patients with largely diffuse and attached lesions that exhibit chaotic motion trajectories, as shown in Figure 5B.

In general, motion-managed PET/CT acquisitions either consist of a small percentage of total detected events in the gating window or a division of total detections across the respiratory states. Either case necessitates increased detection sensitivity to achieve similar signal-to-noise or contrast-to-noise ratios as in motion-free static images. PET acquisitions in 3D mode (lead septa retracted) have been shown to have higher noise-equivalent count rates for clinical injected activities of ^18^ F]FDG (5–10 mCi) than in 2D mode (lead septa inserted) [77]. 3D PET acquisitions are therefore potentially advantageous for respiratory motion-managed image formations.

Image reconstruction considerations depend on the signal-to-noise and contrast-to-noise properties of the motion-managed image acquisition technique. Given the large heterogeneities in thoracic tissues that can impact CT-based attenuation correction of PET activity concentrations, iterative PET reconstructions based on expectation maximization or maximum likelihood tend to produce fewer streak artifacts than those based on analytic filtered backprojection for lung cancer patient imaging studies. Image filtration during or post reconstruction reduces high spatial frequency noise but also worsens resolution of respiratory-induced tumor motion.

As the trend towards complex statistical modeling of imaging systems grows, what is necessary to standardize motion managed PET/CT acquisition and reconstruction protocols across institutions worldwide for clinical translation?

#### Target volume and prescription definition

Definitions of target volumes on reconstructed PET/CT images can be standardized to encompass the residual uncertainty due to respiratory motion within any of the management strategies during image acquisition [78]. One method to define the target and its complete motion envelope has been suggested by Bettinardi and colleagues, whereby they systematically expanded the set of gross tumor volumes (GTV) to internal target volumes (ITV) defined on maximum intensity projections (MIP) across all breathing phases of the respiratory motion-tracked CT and PET images and subsequently calculated the union of the MIP-defined ITVs [79]. This initially led to significantly larger target volumes as compared to those defined on static PET/CT images. However, the expansion of static PET/CT-defined volumes with population-based motion uncertainty margins reduces the difference with patient-specific MIP PET/CT-defined volumes.

Motion-compensated target volumes can be defined within a fixed quiescent gating period or across all respiratory motion-tracked periods. Manual segmentation of respiratory phase-specific target volumes is cumbersome and ideally should be accomplished with an automated procedure. One example includes target contour definition on a single-phase PET/CT image set that is then propagated to the remaining phases via deformable image registration algorithms [80]. This is achieved with high fidelity using the respiratory motion-tracked CT dataset, which define the deformation fields that are subsequently applied to the corresponding respiratory motion-tracked PET dataset. A second automated PET/CT target definition could involve the implementation of auto-segmentation algorithms to contour each respiratory phase-sorted PET/CT, which would not rely explicitly on deformable registration.

Following target definition under PET/CT guidance and motion management, the target prescription can be defined in a variety of ways. Radiation dose escalation to PET-based subvolumes, so-called dose painting, was proposed conceptually over ten years ago [81] and is now being tested in early phase clinical trials [40]. Aristophanous and colleagues have recently investigated the implementation of dose painting in lung cancer radiotherapy planning using motion-managed PET, which introduces several challenges to prescribing dose to moving tumors, and in some cases, independently moving biological target subvolumes [82]. An alternate prescription definition, dose-painting-by-numbers, involves the direct translation of PET image intensity values to prescribed dose, yielding non-uniform spatial distributions across the target volume [83,84]. The advantage of non-uniform prescriptions in the context of motion management of lung cancer radiotherapy is that uncertainties in the tumor motion and image acquisition propagate on average to smaller errors in a continuously varying planned dose distribution compared to errors from uniform prescriptions with sharp dose boundaries.

Will we translate biological imaging-based prescriptions for clinical radiotherapy from top-down empirical models, bottom-up radiobiological models, or some combination of both?

#### Treatment planning

Motion-managed and PET/CT-guided treatment planning begins with the calculation of radiation dose on the appropriate CT images that best represent the patient anatomy during treatment delivery. For conventional radiotherapy under free breathing conditions and no motion compensation, a slow scan or phase-averaged CT image dataset should be used to simulate the motion-blurred anatomical features and equivalent attenuating properties [41]. For motion-suppressed radiotherapy (e.g. under active breathing control or abdominal compression conditions), a fast helical CT scan may be sufficient to capture small residual motion envelopes [41]. For motion-compensated radiotherapy, a respiratory-gated or correlated CT should be used to represent the patient geometry within the gating window or across individual respiratory states.

Optimization of planned radiation dose distributions can directly compensate for motion-blurred delivery or incorporate motion-managed target volumes. In order to account for degradation of the prescribed dose gradient at the edge of moving target volumes during delivery, patient-specific margins that consist of edge-enhanced dose intensity maps can be constructed [85]. Dose can also be optimized based on an average tumor trajectory calculated from a respiratory motion-tracked CT [86] to yield respiratory phase-adapted treatment plans [87]. Respiratory phase-optimized and volumetrically modulated arc therapy, proposed by Chin and Otto, takes advantage of respiratory motion as an additional degree of freedom to preferentially increase dose during portions of the breathing cycle throughout a continuous treatment delivery when the target is isolated from proximal critical structures. This combination of gating and tracking results in plans that are superior to other static, gated, and tracking-based planning methods [76].

Numerous treatment-planning methods motivate careful patient stratification in a similar manner to PET/CT image acquisition and reconstruction. For static and gated PET/CT acquisitions that yield a single set of ROIs, optimizing target margins for delivery can account for residual tumor motion. On the other hand, motion-tracked PET/CT acquisitions that yield phase-specific ROIs can be utilized in a more complex treatment planning strategy. Planned dose can be optimized to uniformly irradiate a maximum intensity projection of the target volume to a lower dose, which would ensure a minimum level of target coverage that includes microscopic extension of disease. Dose can then be escalated to the phase-specific PET/CT-defined biological target volumes with either equal weighting, or preferentially to a particular phase with unequal weighting. The key to these dose escalation strategies is to maintain a fixed integral dose to organs-at-risk. Furthermore, by explicitly including the impact of tumor motion on radiobiological models of cell survival, planned radiation dose could be optimized to improve radiobiological metrics of treatment plan quality, which include tumor control probability, normal tissue complication probability, and generalized equivalent uniform dose [88].

What clinical role will multi-objective optimization play in selecting for treatment plans that minimize dosimetric uncertainties from various motion management strategies?

#### Quality assurance

Residual uncertainties in the estimation of tumor motion during suppressive or compensatory imaging acquisition techniques [89] can be propagated to target volumes, treatment plans, and finally to treatment delivery. This is especially important for random errors that cannot be corrected via on-board image guidance prior to or during treatment delivery. To account for these errors in the delivery of the treatment plan, dosimetric measurements in respiratory torso phantoms can be used to simulate delivery of motion uncompensated or compensated radiotherapy under free-breathing conditions, or to simulate residual motion following suppression strategies.

Ideally, patient-specific tumor motion trajectories would drive the phantom during the delivery of the planned radiation intensity map. Measurements with an array of detectors that offer sufficient spatial resolution can then assess the degree to which treatment plans are being delivered both precisely and accurately. Quality assurance tolerance limits can be enacted for each type of delivery, ranging from static radiotherapy to respiratory-gated or tracked radiotherapy. These limits greatly depend on what deviation is deemed to have a significantly negative impact on patient care. In general, complex respiratory motion-tracked and phase-optimized delivery requires tighter margins for error to avoid geographical misses and potential mistreatment that exceeds acceptable levels of normal tissue toxicity.

Will patient-specific quality assurance for motion managed and PET/CT-guided radiotherapy be ultimately conducted in anthropomorphic phantoms or through real-time dose reconstruction for each treatment fraction?

#### Image-guided treatment delivery

Daily image guidance is essential during delivery of motion-managed and PET/CT-guided radiotherapy of lung cancer. On-board imaging accounts for systematic variations in patient setup, tumor position and motion trajectory under static, gated, or motion-tracked delivery.

Static or gated radiotherapy delivery under active breathing control or abdominal compression relies on careful verification of tumor position and its residual motion envelope from respiratory motion-tracked cone-beam CT (CBCT). The goal is simply to ensure that the motion-suppressed target falls within the planning target volume (PTV) margins throughout the treatment course, shown for an example patient in Figure 4. In the case of gated radiotherapy under active breathing control, the radiation beam would be automatically turned on when the ABC spirometer valve is closed by the patient for a breath hold and turned off when the valve is opened for free breathing.

Respiratory-gated radiotherapy delivery with external respiratory motion surrogates involves a less direct procedure. For example, the daily RPM-defined gated delivery window needs to be matched to the original RPM-defined gated PET/CT imaging window. The gated delivery window would then be verified independently against a respiratory motion-tracked CBCT-defined, so that the internal tumor position falls within the motion-compensated target volume throughout the treatment course. As with any gated delivery, the radiation beam on/off position would be coupled to the RPM system that controls when the respiratory state resides within the gating window.

Respiratory motion-tracked radiotherapy delivery across all respiratory states requires precise tracking of internal fiducials or external respiratory surrogates. The external motion trajectory of these surrogates must correlate to the internal tumor motion trajectory on a per-patient basis at the time of delivery. Under predictive couch tracking of respiratory motion, the time-dependent tumor position as measured by respiratory motion-tracked CBCT must be fixed from the beam’s eye view and fall within the motion-compensated PTV margins. Under predictive multileaf collimator beam tracking of tumor motion, the CBCT-defined tumor trajectory must correlate to the motion-adaptive set of PTVs over all breathing phases.

Daily dose delivery verification during each treatment fraction may eventually be possible with the electronic portal imaging devices (EPID) [90]. These detector arrays can quantify the spatial distribution and magnitude of the exit radiation beam energy flux that has not been attenuated within the patient, which combined with knowledge of the beam’s entrance characteristics and patient’s attenuation map relates an estimate of the patient’s absorbed dose. This form of dose reconstruction, if properly calibrated to absolute dosimeters, could provide a more direct estimate of dosimetric errors in the context of motion-managed radiotherapy.

How do we integrate improved motion tracking and dose verification detector systems with fast dose computation on graphical processor units (GPU) for real-time adaptive treatment delivery?

#### Adaptive procedures

Changes in tumor mass, morphology, and molecular phenotype heterogeneity over the course of treatment can greatly impact the effectiveness of motion-managed and PET/CT-guided radiotherapy. Tumor shrinkage and anatomical deformations can drastically alter the internal motion trajectory, which consequently may no longer correlate to prior trajectories and respiratory surrogate signals. An initial large upper lobe lesion that is attached to the chest wall may not require the same type of motion management as a residual lesion that has detached during the treatment course (ref here).

Daily four-dimensional imaging with CT, typically kilovoltage cone beam CT, enables the calculation of dose on the most current representation of patient anatomy and aides in the decision of whether to adapt the treatment plan to both changes in tumor morphology and trajectory. Furthermore, these changes will likely affect PET avidity and degree of heterogeneity, which can impact biological target definition. However, adaptive radiotherapy based on FDG PET must be approached cautiously due to false positive signal from radiation-induced tissue inflammation, which may be further contaminated by residual motion.

What action threshold criteria should be adopted in adaptive radiotherapy to conform to changing morphology, molecular phenotype, and respiratory-induced tumor motion patterns?

## Conclusions

Advances in lung cancer radiotherapy under FDG PET/CT guidance and respiratory-induced tumor motion management are numerous and approaches are increasingly complex. Molecular imaging continues to impact diagnosis and staging of lung cancer, but refinements in target volume definition and eventually prescribed dose definition are needed. Tumor and nodal motion is accounted for in radiotherapy planning and delivery with uniform spatial margins and daily image guidance, but in the future could be suppressed or compensated for throughout treatment simulation imaging, planning, and delivery. The greatest challenge to the clinical application of these advancements remains the ability to effectively tailor therapeutic strategies to individual patients within a highly heterogeneous population. Identifying patients who will benefit from respiratory motion-tracked radiotherapy versus those who will benefit from respiratory-gated radiotherapy under PET/CT guidance should continue to be a high priority within the radiation oncology research community. The eventual delivery of conformal and escalated radiation dose, either to motion-suppressed or motion-compensated biological target volumes, holds great promise to increase local control rates of lung cancer, reduce normal tissue complication rates, and consequently improve patient outcomes.

## Competing interests

The authors declare that they have no competing interests.

## Authors’ contributions

SB performed the literature review, generated the figures and drafted the manuscript. MN provided reference material and critical revisions to manuscript drafts. MG added clinical introduction and comments to manuscript drafts. KH generated figures and revised manuscript drafts. PK and GS helped to conceptualize structure of manuscript and critiqued technical elements of manuscript drafts. SP added clinical introduction and critiqued clinical elements of manuscript drafts. All authors read and approved the final manuscript.
